# Unmasking Budgerigar Splenic Leukocyte Populations With Single‐Cell Transcriptomics and Multiplex RNA In Situ Hybridization

**DOI:** 10.1155/jimr/1554863

**Published:** 2025-10-09

**Authors:** Anne De Meyst, Kelly Lemeire, Amanda Gonçalves, Niels Vandamme, Daisy Vanrompay

**Affiliations:** ^1^ Laboratory of Immunology and Animal Biotechnology, Department of Animal Sciences and Aquatic Ecology, Faculty of Bioscience Engineering, Ghent University, Ghent, Belgium, ugent.be; ^2^ VIB-UGhent Center for Inflammation Research, Technologiepark-Zwijnaarde, Ghent, Belgium; ^3^ Department of Biomedical Molecular Biology, Faculty of Science, Ghent University, Ghent, Belgium, ugent.be; ^4^ VIB BioImaging Core, VIB-UGhent Center for Inflammation Research, Technologiepark-Zwijnaarde, Ghent, Belgium; ^5^ VIB Single Cell Core, VIB-UGhent Center for Inflammation Research, Technologiepark-Zwijnaarde, Ghent, Belgium

**Keywords:** avian, immune system, *Psittaciformes*, transcriptomics

## Abstract

The current understanding of the avian immune system primarily stems from research conducted in chickens, given their economic significance as a food source. Extending the research to other avian species like *Psittaciformes* requires the use of label‐free techniques. Therefore, budgerigar (*Melopsittacus undulatus*) splenic leukocytes were characterized in this study, with the help of an immunological toolbox, integrating single‐cell transcriptomics and multiplex RNA in situ hybridization (ISH). Twenty‐four distinct psittacine splenic leukocyte populations were identified and characterized, including, amongst others, germinal center (GC) B cells and regulatory T cells (Tregs). For each of these 24 populations, markers were defined for subsequent use in immunological assays. To further examine splenic organization, multiplex RNA ISH was applied, successfully characterizing six out of the nine selected markers. This study showed that the psittacine immune system closely mirrors that of chickens. However, a detailed, comprehensive examination was hindered by the lack of a complete sequenced and annotated budgerigar genome and the limited number of replicates. Consequently, further investigation is imperative to advance our understanding of the avian immune system.

## 1. Introduction

The last common reptilian ancestor of birds and mammals was estimated to have lived around 318 million years ago, marking an important time point in evolutionary history. Despite their early divergence, these two classes of animals share many similarities in their defense mechanisms against pathogens. Both avians and mammals possess primary and secondary lymphoid organs and rely on the coordinated interplay of the innate and adaptive immune response to establish an immunological memory. However, their immune system also differs in some crucial aspects. For instance, birds only possess a rudimentary lymphatic system and lack true lymph nodes, rendering the spleen the most important secondary lymphoid organ for antigen presentation [1]. They also possess a specialized organ for B cell development, the bursa of Fabricius, whereas for mammals this takes place in the bone marrow [2].

The majority of research on the avian immune system has been conducted on domestic chickens (*Gallus gallus*), as these animals are of economic significance in the food industry [3]. Nevertheless, studies on other birds, including turkeys [4], ducks [5], doves [6], guinea fowl [7, 8], and quails [9, 10], have revealed that within birds, the immune system is remarkably similar. The avian immune system comprises two primary lymphoid organs, the thymus and the bursa of Fabricius. These are located respectively alongside the left jugular vein and near the cloaca [11, 12]. The mucosal‐associated lymphoid tissue (MALT) and the spleen are secondary lymphoid organs and play an important role in antigen presentation. In contrast to mammalian spleens, the avian spleen has a closed circulation, characterized by the direct connection of penicillar capillaries with venous sinuses, reducing its role in the filtration of senescent erythrocytes [1]. Overall, the basic structure of the avian spleen is concordant with that of mammals. It is surrounded by a thin collagen capsule, which contains reticular fibers entering the splenic tissue. The spleen is further divided into red pulp and white pulp areas, although the distinction between both is in most birds not as clearly defined as in mammals [6]. The red pulp filters out circulating senescent erythrocytes and contains CD8^+^ T cells and plasma cells near the large blood vessels, along with nonlymphoid cells such as heterophils and red pulp macrophages. Heterophils are the most abundant granulocytic leukocytes in birds and are considered the functional equivalent of mammalian neutrophils. In avian splenic white pulp, three distinct areas are identified: (1) the periarteriolar lymphoid sheath (PALS), (2) the periellipsoid white pulp (PWP), and (3) the germinal centers (GCs) housing B cells and follicular dendritic cells (DCs), developing at the origin of the PALS. The PALS is located around central arteries and is characterized by the presence of CD4^+^ T cells and interdigitating DCs. Penicillar capillaries are surrounded by ellipsoids or Schweigger–Seidel sheaths, containing one to five concentric layers of oval or round reticular cells. The PWP is located around these ellipsoids and forms the counterpart of the mammalian marginal zone. In this zone, B cells are surrounded by a ring of macrophages [1, 10, 13, 14].

Compared to other bird species, little is known about the immune system of *Psittaciformes*. Despite their lack of economic significance in the food industry, *Psittaciformes* represent a substantial share of the pet sector. The imperative to develop targeted therapeutics for these avian companions necessitates an in‐depth understanding of their immune system. In this study, we therefore established an immunological toolbox to study their immune system, employing molecular techniques such as single‐cell transcriptomics (scRNA seq) and multiplex RNA in situ hybridization (ISH). With these innovative technologies, splenic leukocyte populations were identified in psittacine birds, and population markers were determined. Lastly, the morphology of the psittacine spleen was examined, contributing to a better understanding of their immune physiology.

## 2. Materials and Methods

**Figure 1:**
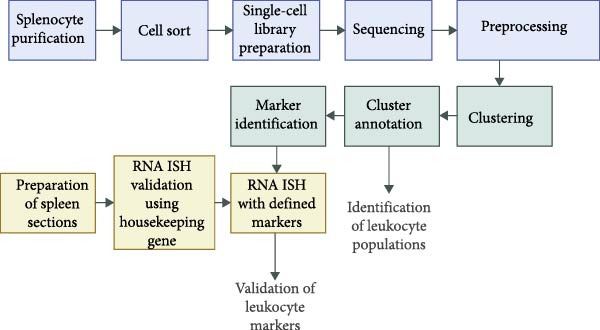
Workflow of immunological toolbox using scRNA seq and multiplex RNA‐ISH (created with Biorender.com).

To gain insight into splenic leukocyte populations of budgerigars, an immunological toolbox was established, combining scRNA seq and RNA‐ISH. An overview of the workflow is shown in Figure 1.

### 2.1. Single‐Cell RNA Sequencing

#### 2.1.1. Animals

All birds used in this study were purchased from a local pet shop. For scRNA seq, one adult female budgerigar (*Melopsittacus undulatus*) was used. For RNA‐ISH analysis, three adult birds (two males, one female) were used. Age was estimated between 6 and 24 months based on beak and cere characteristics. None of the birds were vaccinated or in a reproductive state. Immediately after purchase, the birds were euthanized using a 2‐phase CO_2_/O_2_ system. In a first stage, animals were anesthetized with a low CO_2_ and increased O_2_ concentration (40% CO_2_ and 28% O_2_ mixed with air during 1 min; reversible anesthesia) and then sufficiently anesthetized in a high CO_2_ concentration (60% to 80% CO_2_ mixed with air, during 2 min; irreversible anesthesia), followed by cutting the jugular vein. The study was approved by the Ethical Committee for Animal Experiments of Ghent University (EC‐2019/086).

#### 2.1.2. Isolation of Budgerigar Splenocytes

**Figure 2:**
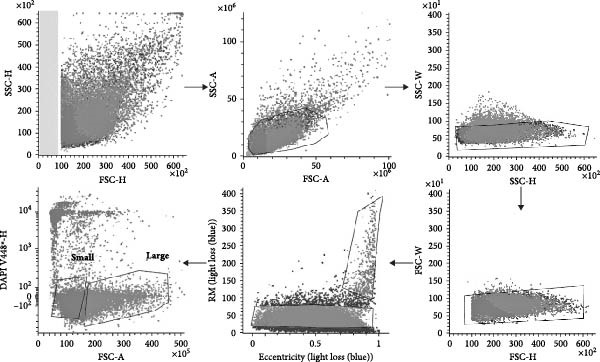
Gating strategy for isolation of budgerigar splenocytes prior to scRNA seq analysis.

Following euthanasia of a single budgerigar, the spleen was immediately isolated and placed in 5 mL Hanks’ balanced salt solution (HBSS; Gibco, Paisley, UK), supplemented with 10% fetal bovine serum (FCS; Greiner Bio‐One, Vilvoorde, Belgium) at RT. An enzymatic digest was conducted for 20 min at 37°C and 1200 RPM using an orbital shaker (Thermal Shake lite, Avantor, Leuven, Belgium), in the presence of 0.5 mL RPMI 1640 medium (Gibco), 0.4% DNase (10 U/µL; Roche, Machelen, Belgium), and 2% Liberase (1 mg/mL; Roche). The resulting cell suspension was filtered through a 70 µm cell strainer and washed with HBSS at RT. Next, splenocytes were isolated through density gradient centrifugation, where 2 mL cell suspension was carefully layered onto 2 mL Lymphoprep (density: 1.077 g/mL; STEMCELL technologies, Geel, Belgium) and centrifuged for 20 min at 800 × *g* and 18°C. This method selectively enriches mononuclear cells while effectively removing red blood cells and a large part of the granulocytes. Cells recovered from the HBSS–Lymphoprep interface were then washed with HBSS (RT), stained with DAPI (10 µg/ml; Invitrogen, Carlsbad, California, USA), and subjected to cell sorting. Images were captured using a BD Biosciences FACS imaging‐enabled prototype cell sorter,equipped with an optical module allowing multicolor fluorescence imaging of fast‐flowing cells in a stream, enabled by BD CellViewTM Image Technology based on fluorescence imaging with radiofrequency‐tagged emission (FIRE) using a BDFacsVulcan (BD Biosciences, Erembodegem, Belgium) [15]. Dead cells and cell aggregates were excluded, and a total of 100,000 small‐sized and 50,000 large‐sized cells were sorted together for further analysis. The sorting strategy is visualized in Figure 2.

#### 2.1.3. Preprocessing and Quality Control

All sorted cells were loaded onto one Chromium Single Cell Chip G (10X Genomics, Leiden, The Netherlands) for encapsulation, capturing, and barcoding of single cells in gel bead‐in‐emulsions (GEMs), using the Chromium Single Cell 3^′^ GEM library and Gel Bead kit V3.1 (10X Genomics). Next, reverse transcription, cDNA amplification, and expression library construction were performed according to the manufacturer’s guidelines. Libraries were sequenced with an Illumina NovaSeq 6000 instrument (Illumina, Mechelen, Belgium), attaining transcripts from 12,843 cells with an average of 23,692 reads per cell and a sequencing saturation of 53.5%. The FastQ file was converted into a count matrix with Cellranger‐5.0.0, and reads were aligned to the reference genome of the budgerigar (*Melopsittacus_undulatus*_6.3: GCA_000238935.1). The resulting unique molecular identifier (UMI) count matrix was imported into R version 4.1.2 and further analyzed with the Seurat package V4.1 [16]. First, the count matrix was filtered by removing genes expressed in less than 3 cells and cells expressing fewer than 200 genes. Secondly, the data were preprocessed with the Scater package [17]. The count matrix was therefore converted into a SingleCellExperiment object, and quality control metrics were calculated for each cell with the addPerCellQC() function. Outliers were detected with the isOutlier() function, which identifies cells with extreme log‐library sizes or an extreme percentage of mitochondrial genes.

#### 2.1.4. Clustering and Identification of Cell Type Markers

After removal of these outliers, a Seurat object was created, and counts were normalized by a log‐transformation using the NormalizeData() function. Subsequently, a scaling was conducted with the ScaleData() function on the 2000 most variable genes, which were identified with the FindVariableFeatures() function. Afterwards, a principal component analysis (PCA) was executed, and an elbow plot was generated with the ElbowPlot() function, illustrating the number of principal components against the percentage of variance explained by each component. The plot indicated that the first 25 principal components contained almost all relevant information, leading to the decision to retain only these components for the clustering analysis. Clustering was performed using the FindNeighbours() function, which automatically generates a matrix containing a *k*‐nearest neighbor (kNN) graph and a shared nearest neighbor (SNN) graph. Subsequently, the Louvain algorithm was applied to identify clusters based on the SNN neighbor information, utilizing the FindClusters() function with a resolution set at 0.8. This resolution was selected because higher resolutions did not yield additional biologically relevant clusters. Visualization of clusters after dimension reduction was achieved through both uniform manifold approximation and projection (UMAP) and *t*‐distributed stochastic neighbor embedding (*t*‐SNE) plotting, generated using the Dimplot() function. Doublets were detected by the DoubletFinder package but were not removed from the analysis [18].

Next, differentially expressed (DE) genes were detected with the FindAllMarkers() and FindMarkers() functions. All clusters were classified into broad populations, with additional refinement where feasible, using a combination of literature and gene databases. Lastly, marker genes were selected that identify these populations. Feature plots, heatmaps, and ridge plots were constructed with the ggplot2 package v3.3.5 and the ggpubr package [19].

### 2.2. RNA ISH

#### 2.2.1. Validation of RNAscope With a Housekeeping Gene

Multiplex RNA‐ISH was executed on budgerigar spleen tissue using the RNAscope technology to validate markers selected with scRNA seq. This technology was first validated on budgerigar spleen tissue with the RNAscope 2.5 HD Red Assay (Advanced Cell Diagnostics Inc., Abingdon, United Kingdom), targeting the housekeeping gene ribosomal protein S7 (*RPS7*). A negative control was included as well, targeting the bacterial L‐2,3‐dihydrodipicolinate reductase (*dapB*) gene. Briefly, three budgerigars were sacrificed as mentioned above, and spleens were isolated and fixed for 16 h at RT in 10% phosphate‐buffered formalin (Sigma–Aldrich, Hoeilaart, Belgium). Afterwards, formalin‐fixed paraffin‐embedded (FFPE) tissue sections were prepared and pretreated by deparaffinization, followed by target retrieval (20 min) and protease treatment (15 min), according to manufacturer’s guidelines. Afterwards, tissue sections were stained for 2 h at 40°C with a custom‐developed probe (sequence confidential to Biotechne), targeting budgerigar *RPS7* (XM_005153227.4), followed by six rounds of signal amplification, again following the manufacturer’s guidelines. Lastly, tissue sections were counterstained with Hematoxylin Gill III (Sigma–Aldrich), air‐dried for 30 min at 60°C, and mounted with EcoMount mounting fluid (Advanced Cell Diagnostics Inc.). Visualization of tissue sections was performed with the ZEISS Axioscan Z1 (Carl ZEISS NV, Zaventem, Belgium), and images were processed with ZEN 3.5 (Blue edition).

#### 2.2.2. Hiplex RNAscope

After validation of the RNAscope technique on FFPE spleen tissue, probes were custom‐designed (sequences confidential to Biotechne), targeting the following leukocyte markers: BCL11A (accession number XM_005143794.3; B‐cell marker), TXNDC5 (accession number XM_034061381.1; plasma cell marker), UHRF1 (accession number XM_034070862.1; GC B‐cell marker), PLBD1 (accession number XM_005150290.2; macrophage marker), CSF3R (accession number XM_005142552.4; DC marker), FAM183A (accession number XM_005151052.4; natural killer [NK] cell marker), TRA (accession number XM_034070013.1; helper T (Th)‐cell marker), TNFRSF9 (accession number XM_034068384.1; cytotoxic T (Tcyt)‐cell marker), and MENT (accession number XM_005149930.3; heterophil cell marker). ISH to these targets was performed in triplicate with the RNAscope HiPlex v2 Assay (Advanced Cell Diagnostics Inc.). This assay allows the detection of all nine targets within the same tissue through consecutive cycles of probe binding and cleaving. To achieve this, FFPE tissue sections were deparaffinized and pretreated by target retrieval (15 min) and protease treatment (25 min). Subsequently, tissue sections were bleached in antigen‐unmasking solution (Vector Laboratories, Brussels, Belgium) with an LED source of 20,000 LUX placed at a distance of 40 cm for 4 days at 4°C, to reduce tissue autofluorescence (adapted from Sun et al. [20]). After pretreatment, tissue sections were submitted to four consecutive rounds of visualization according to the manufacturer’s guidelines. Table 1 provides a summary of the targets visualized in each respective round.

**Table 1:** Visualization scheme of leukocyte markers during the Hiplex RNAscope assay.

Visualization round		R1	R2	R3	R4
Fluorescent dye	DAPI	DAPI	DAPI	DAPI	DAPI
	AF488	FAM183A	CSF3R	MENT	Background
	Dylight 550	BCL11A	TRA	TXNDC5	Background
	Dylight 650	UHRF1	PLBD1	TNFRSF9	Background

Between each round, slides were mounted with Prolong Gold Antifade Mountant (Life Technologies, Geel, Belgium) and covered with a coverslip. Overview images were taken with a Leica microscope DMi 8 (Leica MicroSystems, Diegem, Belgium) using a 200 × magnification and an exposure time of 300 ms for the AF488, Dylight 550, and Dylight 650 channels, and an exposure time of 50 ms for the DAPI channel. Individual TIF files were created from every channel in every round with LASX Office 1.4.5. Lastly, RNAscope Hiplex image registration software v2.1 was used for registration, merging, background removal, and visualization of all nine fluorescent images simultaneously. After all visualization rounds, sections were additionally stained with hematoxylin (2 min; Sigma–Aldrich) and eosin (1 min; Sigma–Aldrich) to correlate between morphology and fluorescent images.

## 3. Results and Discussion

Exploring the immune system of unconventional animals presents challenges, particularly when species‐specific immunological assays are unavailable. The immune system of psittacine birds remains largely unstudied, relying only on extrapolation of knowledge from economically more relevant species, such as chickens. To gain insight into the immune system of psittacines, we initially examined cross‐reactivity of commercially available antichicken antibodies with psittacine leukocytes. Unfortunately, this approach yielded limited success (unpublished data), prompting the exploration of alternative label‐free technologies. This study introduces an immunological toolbox that integrates scRNA seq and multiplex RNA‐ISH to address these challenges. The toolbox facilitates the identification and characterization of immune cell populations and allows the selection and validation of cell type markers. As the spleen is the primary location for antigen presentation and the most important secondary lymphoid organ in birds, the immunological toolbox was applied to budgerigar splenocytes.

### 3.1. Identification of Splenic Leukocyte Populations in Budgerigars

#### 3.1.1. Broad Annotation

In order to classify the 24 cell type clusters into broad populations, DE genes were determined for each cluster by comparing the expression in that cluster to all remaining cells. The 20 most DE genes were then defined as the genes with the highest absolute values of log fold change (FC) and are compiled in Table 2. Gene abbreviations can be found in Table S1. Notably, a substantial number of genes remained unannotated following alignment with the budgerigar reference genome, likely due to the genome’s incomplete annotation. To address this limitation, genes that exhibited interesting characteristics, such as high log FC or specific expression patterns, but lacked official annotation, were further investigated using BLAST searches to infer their identity based on sequence homology. When a gene was identified via BLAST analysis, this was consistently indicated either in the main text or in the corresponding tables.

**Table 2:** The 20 most differentially expressed genes in all cell type clusters together with their annotation into broad populations.

Cell type cluster	Annotation	Differentially expressed genes
0	B lymphocytes	Up: *RPS12*, *RPS20*, *HVCN1* Down: *LCN1*, *MRP126* ^∗^, *JCHAIN*, *IGLL1*, *SAA1*, *CD3*ε, *CXCL8*, *TRA* ^∗^, *DUSP1*, *CD3*γ, *CST3*, *CTSD*, *TRB* ^∗^, *CCL4*, *ID2*, *TRD* ^∗^, *SPINK2* ^∗^

1	T lymphocytes	Up: *TRA* ^∗^, *TRB* ^∗^, *CD3*γ, *CD3*ε, *TSC22D3*, *FXYD6*, *AKR1D1* Down: *CCL4*, *LYN*, *PSAP*, *IFI27L2*, *HVCN1*, *CXCL8*, *SAA1*, *FTH1*, *MRP126* ^∗^, *MHC-DRA*, *IGLL1*, *CD74*, *LCN1*

2	B lymphocytes	Up: *HVCN1*, *CXCR4*, *PIK3AP1*, *MYC*, *ENSMUNG00000008668* ^∗∗^, *LYN* Down: *LCN1*, *IGLL1*, *SAA1*, *CD3*ε, *CXCL8*, *TRA* ^∗^, *CD3*γ, *DUSP1*, *CST3*, *CCL4*, *CTSD*, *ID2*, *TRB* ^∗^, *TRD* ^∗^

3	B lymphocytes	Up: ‐ Down: *LCN1*, *MRP126* ^∗^, *JCHAIN*, *IGLL1*, *SAA1*, *RPL35*, *FTH1*, *RPLP2*, *EEF1A1*, *RPL26*, *RPL30*, *RPS15A*, *TPT1*, *RPS29*, *RPL27A*, *RPS20*, *RPL21*, *RPL27*, *RPS8*, *RPS25* ^∗^

4	T lymphocytes	Up: *CD3*γ, *CD3*ε, *IL18R1*, *TNFRSF18*, *TRB* ^∗^, *DUSP1*, *I*COS, *SRSF7*, *TRA* ^∗^, *PIK3R1* Down: *IGLL1*, *MRP126* ^∗^, *MHC-DRA*, *CD74*, *SAA1*, *FTH1*, *PLAC8*, *IFI27L2*, *HVCN1*, *CXCL8*

5	B lymphocytes	Up: *IFI27L2*, *PLAC8*, *HVCN1*, *OASL*, *CXCR4*, *IFIT5* ^∗^ Down: *JCHAIN*, *IGLL1*, *CD3*ε, *CXCL8*, *CD3*γ, *DUSP1*, *TRA* ^∗^, *CTSD*, *ID2*, *CCL4*, *TRD* ^∗^, *TRB* ^∗^, *LSP1*, *TSC22D3*

6	B lymphocytes	Up: *JCHAIN*, *IGLL1*, *HSP90B1*, *TXNDC5*, *PPIB*, *HSPA5*, *RBM15B* Down: *LCN1*, *BTG1*, *ZFP36L1*, *CXCR4*, *MRP126* ^∗^, *ACTB*, *BTG2*, *ACTG1*, *FTH1*, *PLAC8*, *HSPA8*, *KLF2*, *SAA1*

7	Mononuclear phagocytes	Up: *SAA1*, *CST3*, *MHC-DRA*, *C1QA*, *IFI27L2*, *MARCO*, *FTH1*, *HMOX1*, *CXCL8*, *CD74*, *CCL4*, *CTSD*, *PSAP*, *MHC-DRB1* ^∗^, *SAT1*, *C1QC*, *CR1* Down: *MRP126* ^∗^, *IGLL1*, *TRB* ^∗^

8	T lymphocytes	Up: *BCL11B*, *ND6*, *TRB* ^∗^ Down: *LCN1*, *JCHAIN*, *SAA1*, *CXCL8*, *FTH1*, *MHC-DRA*, *CD74*, *CST3*, *CCL4*, *CTSD*, *IFI27L2*, *LGALS1*, *PRDX1*, *HMOX1*, *LY6E*, *MHC-DRB1* ^∗^, *ANXA2*

9	T lymphocytes	Up: *TRD* ^∗^, *XCL1*, *TNFRSF9*, *CD9*, *CD3*ε, *ID2*, *IGKC* ^∗^, *ENSMUNG00000001681* ^∗∗^, *RB1CC1*, *LONRF3*, *CPE* Down: *LCN1*, *IGLL1*, *JCHAIN*, *FTH1*, *SAA1*, *CD74*, *MHC-DRA*, *CXCR4*, *CXCL8*

10	Mononuclear phagocytes	Up: *CTSD*, *IFITM5*, *SAA1*, *FTH1*, *HMOX1*, *MHC-DRA*, *B2M*, *ANXA2*, *TXN*, *PRDX1*, *PSAP*, *S100A11*, *CD74*, *SQSTM1*, *PLBD1*, *CSRP1* Down: *IGLL1*, *MRP126* ^∗^, *JCHAIN*, *BTG1* ^∗^

11	B lymphocytes	Up: *STMN1*, *H1*, *TOP2A*, *NUSAP1*, *SMC2*, *MKI67* ^∗^, *TUBB4B*, *HMGB2*, *H2AZ1*, *H1-3*, *HMGB1*, *UHRF1*, *YBX1*, *H1-10*, *KIF11*, *UBE2C*, *CKAP2* Down: *LCN1*, *MRP126* ^∗^, *JCHAIN*

12	T lymphocytes	Up: *TRD* ^∗^, *ENSMUNG00000001354* ^∗∗^, *CD3*γ, *CD3*ε, *FXYD6*, *TSC22D3*, *HSPA2* Down: *LCN1*, *IGLL1*, *CD74*, *MHC-DRA*, *SAA1*, *FTH1*, *CXCL8*, *HVCN1*, *IFI27L2*, *LYN*, *PSAP*, *CCL4*, *MHC-DRB1* ^∗^

13	Other leukocytes	Up: *MRP126* ^∗^, *LCN1*, *Avidin*, *MENT*, *Caltrin2* ^∗^, *LTF*, *RGS2*, *CXCL8*, *CSF3R*, *F10*, *ID2*, *CES3* Down: *IGLL1*, *MHC-DRA*, *ND3*, *CD74*, *RPS12*, *JCHAIN*, *ND6*, *RPS29*

14	B lymphocytes	Up: *ND5*, *ND4* Down: *LCN1*, *IGLL1*, *RPL30*, *EEF1A1*, *RPLP2*, *RPL26*, *RPS15A*, *RPL35*, *TPT1*, *RPL27A*, *RPL21*, *RPL29*, *RPS8*, *RPS25* ^∗^, *RPL27*, *RPS3*, *RPS3A*, *RPS27A*

15	Mononuclear phagocytes	Up: *LCN1*, *CXCL8*, *SAA1*, *LY6E*, *CCL4*, *S100A11*, *ANXA2*, *CRIP1*, *CSF3R*, *GLUL*, *LY86*, *LGALS1*, *Avidin*, *FTH1*, *MRP126* ^∗^, *PRDX1*, *VIM*, *CFH* Down: *DDIT4*, *TRB* ^∗^

16	Other leukocytes	Up: *APOA1*, *CFD*, *GST*, *PPDPF*, *PENK*, *CKB*, *CLU*, *IFI27* ^∗^, *EPB41L2*, *HSPA*, *UCHL1*, *ATP6*, *RTN4*, *GST*, *TXNIP* Down: *IGLL1*, *CD74*, *MHC-DRA*, *CXCR4*, *SAA1*

17	B lymphocytes	Up: ‐ Down: *ENSMUNG00000000005* ^∗∗^, *COX1*, *COX3*, *COX2*, *ND3*, *ENSMUNG00000004039* ^∗∗^, *ENSMUNG00000005153* ^∗∗^, *LCN1*, *ENSMUNG00000000003* ^∗∗^, *MRP126* ^∗^, *HSP90AA1*, *TRA* ^∗^, *ND6*, *CYTB*, *ND4*, *ND5*, *SAA1*, *ENSMUNG00000008668* ^∗∗^, *AKAP9*, *KMT2C*

18	Other leukocytes	Up: *SPINK2* ^∗^, *TPPP2*, *CBY2*, *FAM183A*, *TSSK6*, *REEP6*, *VTN*, *TEX36*, *TUBA2*, *JPT1*, *SPACA9*, *NDUFAF4*, *ENSMUNG00000016316* ^∗∗^, *SCCPDH*, *ENSMUNG00000013590* ^∗∗^, *ODF2*, *TRAF3IP1*, *NRGN*, *NME8*, *PACRG* Down: ‐

19	T lymphocytes	Up: *TRA* ^∗^, *IFI27L2*, *MX1*, *IFIT5* ^∗^, *TRB* ^∗^, *OASL*, *STAT1*, *SAMD9L*, *CD3*γ, *PLA2G4C*, *PLAC8*, *FXYD6* Down: *IGLL1*, *CD74*, *MHC-DRA*, *FTH1*, *PSAP*, *LYN*, *HVCN1*, *MHC-DRB1* ^∗^

20	B lymphocytes	Up: *CCL4*, *BASP1*, *PKM*, *HVCN1*, *NPM1*, *RPS20*, *DOK3*, *RPS12*, *HIVEP3* Down: *JCHAIN*, *CD3*ε, *IGLL1*, *CXCL8*, *TRA* ^∗^, *CD3*γ, *TRB* ^∗^, *DUSP1*, *TSC22D3*, *TRD* ^∗^, *LSP1*

21	Mononuclear phagocytes	Up: *CST3*, *CR1*, *MARCO*, *DNASE1L3*, *VCAM1*, *TGFBI*, *CXCL8*, *LY6E*, *RARRES1*, *PSAP*, *FOS*, *EHD3*, *SAA1*, *SPIC*, *RAB10*, *TPTE2*, *TUBB6*, *CD48* ^∗^, *CCL4* Down: *BTG1*

22	APC	Up: *CXCL8*, *LY86*, *IRF8*, *CTSL* ^∗^, *DNASE1L3*, *IFI27L2*, *JCHAIN*, *MGST3*, *FTH1*, *MX1*, *CD74*, *CR1*, *TNFAIP2*, *FOS*, *DUSP7*, *LY6E* Down: *IGLL1*, *ENSMUNG00000008668* ^∗∗^, *BTG1*, *KLF2*

23	Other leukocytes	Up: *USP44* Down: *CD74*, *RPL35*, *FTH1*, *RPS8*, *MHC-DRA*, *RPS20*, *RPL27A*, *EEF1A1*, *RPL26*, *RPS3A*, *RPLP2*, *TPT1*, *RPS29*, *RPS12*, *RPS15A*, *RPL9*, *RPL35A*, *RPL29*, *RPS25* ^∗^

*Note*:  ^∗^ signifies annotated via BLAST search;  ^∗∗^ signifies noncoding uncharacterized RNA.

Multiple cell type clusters expressed genes from the T‐cell receptor (TCR) complex, including *TRA*, *TRB*, *TRD*, *CD3ε*, and *CD3γ*. According to Smith and Göbel [21], all jawed vertebrates possess four different TCR chains (A, B, C, and D), forming either TCRαβ or TCRγδ heterodimers [21]. While *TRA*, *TRB*, and *TRD* (all three genes identified through BLAST search) were detected in our dataset, *TRC* was notably absent. This absence is likely attributable to the incomplete genome sequencing and gene annotation for budgerigars. The avian CD3 signaling complex, attached to the TCR heterodimer, comprises two genes (*CD3ε* and *CD3γδ*), differing from mammals, where the complex includes CD3ε, CD3γ, and CD3δ [22]. As expected, several cell type clusters in budgerigar splenocytes exhibited differential expression of *CD3ε* and *CD3γ*. The latter is presumably homologous to chicken *CD3γδ*, equally resembling mammalian *CD3γ* and *CD3δ*. Based on the expression pattern of the aforementioned genes, cell clusters 1, 4, 8, 9, 12, and 19 were annotated as T lymphocytes.

Clusters 7 and 10 exhibited high expression of genes homologous to human *MHC-DRA* and *MHC-DRB1*, as identified through BLAST analysis; these correspond to the avian class II MHC genes *B-LA* and *B-LB1*, respectively [23]. Cell type clusters 21 and 22 also significantly upregulated these two genes, but they did not belong to the 20 most DE genes and are therefore not listed in Table 1. While a comprehensive discussion of avian MHC genes is beyond the scope of this study, it is worth noting that both *MHC-DRA* (*B-LA*) and *MHC-DRB1* (*B-LB1*) are part of the MHC Class II complex, typically expressed by antigen‐presenting cells (APCs), with DCs expected to show the highest expression in both mammals and birds [3, 23]. In addition to the elevated MHC Class II expression in clusters 7 and 21, these clusters displayed a significantly higher expression of *MARCO*, compared to other clusters. Given that *MARCO* is a selective marker for innately activated macrophages in mammals, these two clusters were annotated as mononuclear phagocytes [24]. Clusters 10 and 15 were similarly categorized due to their increased expression of *ANXA2* and its ligand *S100A11*, which are highly expressed on the surface of mammalian macrophages, to mediate macrophage activation [25]. The final cell cluster significantly expressing MHC class II genes was cluster 22. While the genes listed in Table 2 did not readily disclose the immune cell type of this cluster, the expression profile suggested that it did not comprise T lymphocytes. The downregulation of *KLF2*, a mammalian regulator of T‐cell quiescence and migration, and the absence of other previously mentioned T‐cell markers, supported this observation [26]. Conversely, the expression of several other genes aligned with the profile of mammalian APCs, including MHC Class II genes, *IRF8* [27], and *CD74* [28]. Therefore, cluster 22 was categorized as APCs pending further analysis.

Identification of B lymphocytes based on the 20 most DE genes appeared challenging. One noteworthy gene expressed across various clusters was the tyrosine‐protein kinase *LYN*. This gene displayed high expression in cell type cluster 2 (Table 2) and moderate expression in clusters 0, 5, and 20. As LYN functions during the initial step of B‐cell receptor signaling in mammals, these four clusters were annotated as B lymphocytes [29]. Since other B‐cell receptor signaling genes were not featured in Table 2, a more thorough analysis of B‐cell receptor genes was undertaken. Only a few genes related to B‐cell receptor signaling were found in the database. For instance, *BTK*, a cytoplasmic protein tyrosine kinase essential for B cell proliferation and survival in avians and mammals, was significantly expressed in cell type clusters 0, 5, and 20, which were already annotated as B lymphocytes [30]. Additionally, *CD79b* (part of the avian and mammalian B‐cell receptor complex) and the tyrosine kinase *Syk* (recruited during BCR signaling in birds and mammals) were also expressed by these three clusters, albeit not differentially [31–33]. Other B‐cell receptor signaling genes were not found in the dataset.

Apart from the expression of B‐cell receptor signaling genes, B cells can also be identified by their expression of immunoglobulin (Ig) genes. Avian antibody development differs markedly from mammals due to their possession of only three antibody isotypes (IgA, IgM, and IgY) and their distinctive way of regulating antibody diversity. In contrast to mammals, birds only have a limited set of variable and joining genes, limiting diversity through gene rearrangement. Chickens, for instance, only possess a single copy of functional variable and joining genes within the gene clusters encoding the light and heavy chains. Conversely, antibody diversity primarily arises through somatic gene conversion, where nonfunctional pseudogenes replace functional copies downstream. Furthermore, avians only possess one light‐chain (*λ*), unlike mammals, which have *λ* and *κ* light chains [34]. A thorough analysis of Ig genes was thereby hampered. Only two genes necessary for antibody production were identified and were strongly upregulated in cluster 6, being *JCHAIN* and *IGLL1*. As these genes are highly expressed by avian and mammalian plasma cells, cluster 6 was identified as a B lymphocyte population [35]. Additionally, cell type cluster 17 also significantly upregulated *IGLL1*, but the gene was not listed in Table 2 as it did not belong to the 20 most DE genes. This cluster further downregulated earlier‐mentioned T‐cell and myeloid cell type markers. Therefore, cluster 17 was also classified as B lymphocytes. The absence of other Ig genes was attributed to incomplete genome sequencing and gene annotation in budgerigars, necessitating further research for a detailed description of antibody response and B‐cell receptor signaling. Next, Table 2 indicates the differential upregulation of *UHRF1* in cluster 11. Given *UHRF1*’s demonstrated critical role in GC B cell proliferation and affinity maturation in mice [36], cell type cluster 11 was consequently annotated as B lymphocytes. Lastly, clusters 3 and 14 were also annotated as B lymphocytes, as these clusters moderately expressed *Pax5*, *EBF1*, and *MEF2C*, three genes strongly associated with B cell development in birds and mammals [37–40]. On the other hand, further analysis will be needed to confirm this hypothesis, as other specific B‐cell markers were absent.

**Figure 3:**
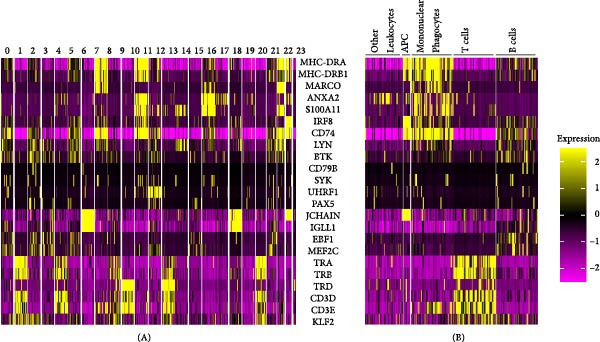
Heatmap of immune cell markers for individual cell type clusters 0–23 (A) and clustered according to the different populations (B).

Given that none of the previously mentioned markers were DE or were downregulated in cell type clusters 13, 16, 18, and 23, except for *ANXA2* and *S100A11* in cluster 16 and *S100A11* and *LYN* in cluster 13, these four populations were provisionally annotated as “other leukocytes,” pending further analysis. A heatmap featuring all above‐mentioned immune cell markers is available in Figure 3. The raw image can be found in Figure S1.

#### 3.1.2. Refined Annotation

The annotated cell type clusters underwent additional subdivision into narrow populations where feasible. Therefore, all cell type clusters within a population were compared to each other, and DE genes were determined. It is important to emphasize that refined annotation of cell type clusters relied on a highly restricted gene set, and further investigation is needed to validate these annotations. Figure 4 presents a conclusive UMAP and *t*‐SNE plot, featuring the final identification of all 24 clusters.

Figure 4(A) UMAP plot and (B) *t*‐SNE plot of cell type clusters. (0) Naïve B cells, (1) naïve or memory Th cells, (2) naïve B cells, (3) memory B cells, (4) regulatory T cells, (5) naïve B cells, (6) plasma cells, (7) M2 macrophages, (8) Th cells, (9) cytotoxic T cells, (10) M1 macrophages, (11) GC B cells, (12) γδ Th cells, (13) heterophils, (14) memory B cells, (15) conventional dendritic cells, (16) CD56^bright^ NK cells, (17) plasma blasts, (18) CD56^dim^ NK cells, (19) Th1 cells, (20) naïve B cells, (21) red pulp macrophages, (22) plasmacytoid dendritic cells, and (23) NKT cells.

**Figure:**
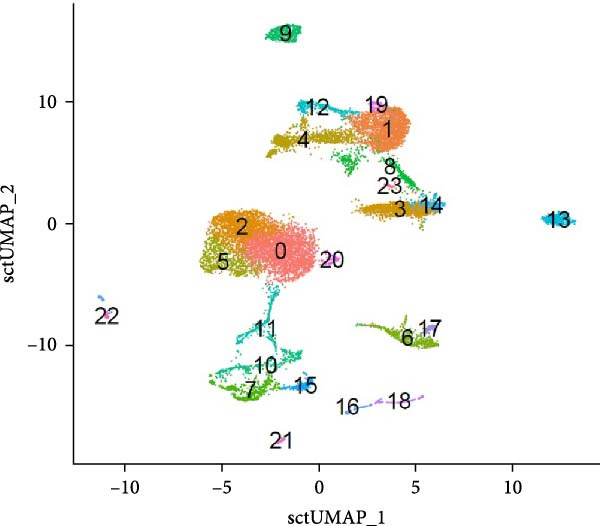
(A)

**Figure:**
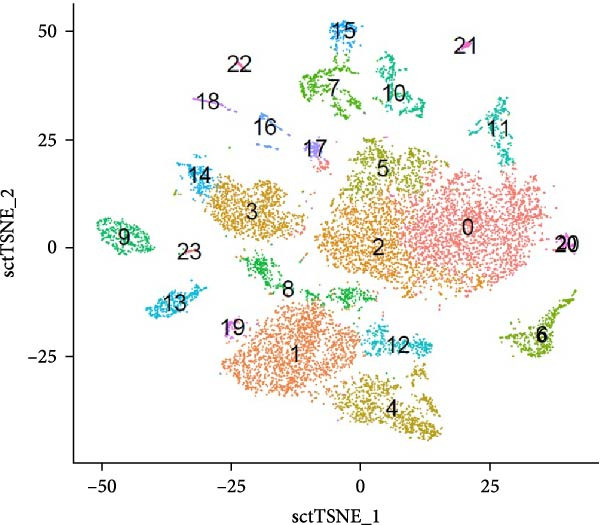
(B)

##### 3.1.2.1. T Lymphocytes

To further annotate T lymphocyte populations, the expression of T‐cell markers *CD4* and *CD8* was examined first. Both *CD8*α and *CD8*β were not DE in any T‐cell population. Notably, *CD8β* was entirely absent from the dataset, presumably again a consequence of incomplete genome annotation. Cluster 9 displayed a faint expression of *CD8*α in less than 5% of its cells, and no expression was observed in other T‐cell populations.

Given the upregulation of *XCL1*, *TNFRSF9*, *IL21R*, and *Eomes*, which are essential Tcyt‐cell genes in mammals, cluster 9 was annotated as Tcyt [41–44]. Interestingly, this population significantly upregulated TRD expression. While most mammalian Tcyt express the TCRαβ heterodimer, birds have been shown to possess a higher frequency of γδ T cells (up to 50%), including CD8^+^ γδ T cells. Even the existence of a splenic γδ T‐cell population with CD8αα homodimers has been reported in young chickens, which might also explain the absence of *CD8*β in the dataset. According to Fenzl et al. [45], chicken γδ T cells represent a major cytotoxic lymphocyte subset that lyses targets in an MHC‐unrestricted manner. This is presumably also the case for psittacines.

*CD4* was also not DE by any T‐cell population and only dimly expressed by less than 5% of the cells from cell type clusters 1, 4, 8, 12, and 19. Given that *CD4* and *CD8* are universal markers for Th cells and Tcyt, respectively, their scarcity in psittacines was surprising. One possible explanation for this finding is that the single‐cell transcriptomics analysis was only executed on one replicate, at one single point in time. Consequently, the observed low expression of these markers might have been merely coincidental. Recently, a study was published where chicken peripheral blood leukocytes were mapped using single‐cell RNA sequencing. This study revealed the expression of CD4 and CD8 genes in multiple T‐cell subsets, but also mentions that caution is necessary when interpreting single‐cell data. A common challenge in leukocyte phenotyping is the low gene expression of key transcripts. Increasing cell numbers and sequencing depth is considered an effective strategy to enhance resolution [46]. In the present study, resolution could be improved, as 12,843 cells were sequenced with a saturation of 53.5%, whereas Maxwell et al. [46] achieved a sequencing saturation between 84% and 91%, albeit with only 4773 cells per sample. Despite this, the consistently low expression levels of CD4 and CD8 remain noteworthy and necessitate further investigation.

Cluster 12 was the second cell cluster, which significantly upregulated TRD. As this cluster also expressed the mammalian Th cell regulator *GATA-3*, this population was annotated as γδ Th cells [47]. In chickens, a new population of cytotoxic innate‐like γδ CD8^-^ T cells was recently described in the intestinal epithelium by [45]. Whether cluster 12 indeed belongs to this cell population is unclear. The function of γδ T cells in birds is not elucidated yet, but as they have a broad tissue distribution, they are suggested to be important for immune surveillance [21, 45]. In cluster 19, multiple genes were upregulated, associated with a mammalian antiviral immune response, including *IFIT5*, *LY6E*, *MX1*, and *IF27L2*. As this population also upregulated *STAT1*, essential for Th1 cell differentiation in mammals, this cluster was annotated as Th1 cells [48]. However, further confirmation is required since other Th1 cell markers were absent in the dataset.

Next to the above‐mentioned T‐cell lineages, Th2 cells, Th17 cells, memory T cells, and regulatory T cells (Tregs) have been described in the chicken. These can be identified by the expression of *IL-4* or *IL-13*, *IL-17*, and *FoxP3*, respectively, but none of these were present in the dataset [49, 50]. On the other hand, cell cluster 4 did upregulate the expression of *IL18R1*, *I*COS, and *CCR6*, all of which are described to be highly expressed by both mammalian Treg and Th17 cells [51–54]. The downregulation of *TCF7* in cluster 4, a characteristic feature of Treg in mice, led to its annotation as Treg [55, 56].

Cell type clusters 1 and 8 were particularly challenging to annotate further. Cluster 1 displayed decreased expression of *TNFRSF18*, a costimulatory receptor associated with mammalian activated T cells, and an increased expression of *TCF7*, commonly associated with naïve or memory Th cells [57, 58]. Just as cell type cluster 1, cluster 8 dimly expressed *CD4* and *GATA-3*, but no further T‐cell markers were found. Therefore, cluster 8 was provisionally annotated as Th cells and cluster 1 as naïve or memory Th cells. Further classification was impaired by the absence of other known T‐cell markers.

##### 3.1.2.2. B Lymphocytes

As mentioned earlier, cluster 11 was the only population with an upregulated expression of *UHRF1* (GC B‐cell marker). Next to *UHRF1*, this cell type cluster also expressed other mammalian cell cycle regulation genes, including *HMGN5*, *SMC2*, and *STMN1* [59]. Consequently, this population was annotated as GC B cells. Unlike mammals, birds form GCs in the T‐cell zone and display a more homogenous structure with a subcapsular dark‐zone homolog and a central light‐zone [38]. The final stage of B cell development involves the formation of plasma cells secreting IgM, IgA, or IgY. *JCHAIN* and *IGLL1*, linked to antibody production, exhibited differential expression in cell clusters 6 and 17. Despite their close association on the UMAP plot, these clusters displayed distinct gene expression profiles. Cluster 17 expressed multiple genes related to mammalian cytoskeletal dynamics, cell cycle regulation, and stress response, including *CCND2* and *BTG1* [59], suggesting active proliferation, while cluster 6 downregulated these genes. We therefore hypothesize that cluster 6 contains fully developed plasma cells, whereas cluster 17 contains actively proliferating preplasma cells or plasma blasts. This hypothesis is further supported by the increased expression of *CREB3L2* in cluster 17, which is strongly induced during the transition from B cell to the plasma cell state in mammals [60].

Other B‐cell populations reported in the avian spleen include naïve B cells, residing in the B cell areas of the spleen, and memory B cells. Until today, follicular and marginal zone B‐cell populations have not been identified in chickens [38]. Two distinct B‐cell populations in the dataset, B cell clusters 0, 2, 5, and 20, and clusters 3 and 14, were compared. Clusters 0, 2, 5, and 20 exhibited higher expression of *RPS11*, *UQCRQ*, *GTPB4*, and *COX7A2*, suggestive in mammals of increased activity [59], along with elevated *MYC* expression, a transcription factor that is transiently expressed in mammals until B cells are fully differentiated into plasma cells or memory B cells [61]. Therefore, clusters 0, 2, 5, and 20 were annotated as naïve B cells. Conversely, clusters 3 and 14, with a more dormant state and lower *MYC* expression, were hypothesized to comprise memory B cells. Further proof to support this hypothesis was not found in the dataset. As populations 0, 2, 5, and 20, and populations 3 and 14 barely differed from each other in gene expression, no further refined annotation was performed.

##### 3.1.2.3. Mononuclear Phagocytes and Other APC

The first cell cluster that could be further annotated was cluster 21. This cluster uniquely expressed *SPIC*, a mammalian transcription factor selectively found in splenic red pulp macrophages. These macrophages play a crucial role in degrading senescent erythrocytes and recycling heme‐associated iron [62].

Cell type clusters 7, 10, and 15 were more closely associated with each other according to the UMAP plot, despite significant variations in gene expression. Cluster 10, for instance, displayed upregulation of *CCR6* and downregulation of *MARCO*, *CD206*, and *C1QA*, *B*, and *C*, while cluster 7 exhibited the opposite pattern. As *CD206* and *C1Q* upregulation is associated with M2 macrophages in mammals, cluster 7 was annotated as M2 macrophages and cluster 10 as M1 macrophages [63, 64]. On the other hand, cluster 15 also upregulated several genes associated with an M2 macrophage profile, but as this population was the only cluster that expressed *DCSTAMP*, a DC‐derived protein in mammals, this population was annotated as conventional DCs (cDCs) [65].

Cluster 22 was more difficult to identify. As mentioned earlier, this population upregulated MHC class II genes and *IRF8*. After comparing its gene expression profile with clusters 7, 10, 15, and 21, cluster 22 appeared to have a higher expression of *TLR3*, *JCHAIN*, *MX1*, and *CD2AP*. In mice and humans, these genes are markers for plasmacytoid DCs (pDCs) [66–68]. However, caution is warranted in classifying cluster 22 as pDCs because these cells have not been identified in avian species yet.

##### 3.1.2.4. Other Leukocyte Populations

Clusters 13, 16, 18, and 23 were initially categorized as “other leukocyte populations” as these clusters lacked the aforementioned T‐cell markers, B‐cell markers, and myeloid cell markers. Clusters 16 and 18 both expressed *NCAM1* (*CD56*) and *Paraoxonase 2*, with cluster 16 displaying the highest expression. As *NCAM1* is a mammalian and avian NK cell marker, and *Paraoxonase 2* is a mammalian NK cell marker, cluster 16 was annotated as CD56^bright^ NK cells and cluster 18 as CD56^dim^ NK cells. Notably, other NK cell markers were absent, including *TRA*, which has been shown to be expressed by chicken NK cells [69]. *TRD* was strongly expressed by cell type cluster 23, together with *RUNX3*, a known mammalian transcription factor in NK cells [70]. Together with the fact that cluster 23 was closely related to T‐cell population 8, this population was provisionally annotated as NKT cells, although further proof was absent. While mammalian NKT cells are characterized by the expression of *TRA* and *TRB*, it is possible that avian NKT cells are *TRD* ^+^, explaining their expression in cluster 23. Nevertheless, this has not been described yet.

Lastly, cluster 13 significantly upregulated expression of *CXCL8*, *HMGB1*, and *SPI1*, three genes associated with mammalian neutrophils [71–73]. As birds do not have neutrophils, cluster 13 was annotated as heterophils, their avian equivalent. It is worth mentioning that a density gradient centrifugation was performed on splenocytes, aimed at removing red blood cells and granulocytes. Although this step greatly reduces their numbers, it does not achieve complete removal. Therefore, the presence of a small population of heterophils is both interesting and anticipated, given their known localization in red pulp sinuses of the avian spleen [1].

### 3.2. Markers of Splenic Leukocyte Populations in Budgerigars

**Figure 5:**
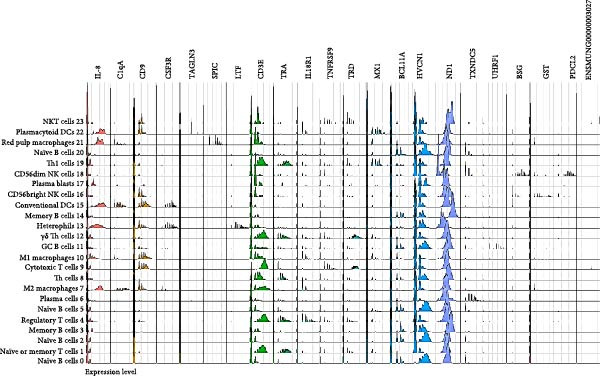
Ridge plot of splenic cell type markers and their cluster‐wise expression levels. The 24 cell type clusters are presented on the *y*‐axis, and the expression level of the markers on the *x*‐axis.

After the creation of a cell‐type annotated reference single‐cell dataset of budgerigar splenocytes, markers were defined for each population. The differential expression metrics are detailed in Table 3, and their expression profiles are visualized in Figure 5. T cells were marked by the distinct expression of *CD3*ε (average log2FC = 2.9683), while Th cells were characterized by *TRA* expression, a deviation from the universal *CD4* marker observed in other animals [21]. As expected, according to expression profiles in mammals, *IL18R1*, *TNFRSF9*, and *TRD* were selected as markers for Treg, Tcyt, and γδ Th cells, respectively [41, 45, 51]. Further, *MX1* was shown to be upregulated in mammalian Th1 cells before, and was also selected in this study as a marker for this population [74].

**Table 3:** Markers of splenic leukocyte populations: marker genes, cell clusters, and differential expression metrics. pct.1 = percentage of cells within cell cluster expressing the marker gene, pct.2 = percentage of cells within all other cell clusters expressing the marker gene.

Cell type (cluster)	Marker gene	Average log_2_FC	pct.1 − pct.2	Adjusted *p*‐value
T cells (1, 4, 8, 9, 12, 19)	*CD3E* (cluster of differentiation 3E)	2.968	0.955 − 0.255	0.00E + 00
Th cells (1, 4, 8, 12, 19)	*TRA* (T‐cell receptor α locus)	4.554	0.877 − 0.126	0.00E + 00
Treg cells (4)	*IL18R1* (interleukin 18 receptor 1)	4.142	0.699 − 0.075	0.00E + 00
Tcyt cells (9)	*TNFRSF9* (tumor necrosis factor receptor superfamily member 9)	5.157	0.824 − 0.063	0.00E + 00
γδ Th cells (12)	*TRD* (T‐cell receptor δ locus)	4.393	0.997 − 0.138	0.00E + 00
Th1 cells (19)	*MX1* (MX dynamin‐like GTPase1)	3.999	0.637 − 0.071	1.17E − 84
B cells (0, 2, 3, 5, 6, 11, 14, 17, 20)	*BCL11A* (BCL11 transcription factor A)	2.865	0.522 − 0.092	0.00E + 00
Naïve B cells (0, 2, 5, 20)	*HVCN1* (hydrogen voltage‐gated channel 1)	2.532	0.971 − 0.331	0.00E + 00
Memory B cells (3, 14)	*ND1* (NADH dehydrogenase 1)	1.522	1.000 − 0.986	0.00E + 00
Plasma cells (6, 17)	*TXNDC5* (thioredoxin domain‐containing 5)	4.454	0.750 − 0.119	0.00E + 00
GC B cells (11)	*UHRF1* (ubiquitin‐like with PHD and ring finger domains 1)	6.368	0.761 − 0.021	0.00E + 00
Phagocytes (7, 10, 13, 15, 21, 22)	*CXCL8* (CXC motif chemokine ligand 8)	6.264	0.785 − 0.117	0.00E + 00
M2 macrophages (7)	*C1QA* (Complement c1q A chain)	6.246	0.852 − 0.043	0.00E + 00
M1 macrophages (10)	*CD9* (cluster of differentiation 9)	3.591	0.913 − 0.127	0.00E + 00
cDC (15)	*CSF3R* (colony stimulating factor 3 receptor)	5.131	0.990 − 0.048	0.00E + 00
pDC (22)	*TAGLN3* (transgelin 3)	8.686	0.560 − 0.001	0.00E + 00
Red pulp macrophages (21)	*SPIC* (spi‐C transcription factor)	10.090	0.915 − 0.002	0.00E + 00
Heterophils (13)	*LTF* (lactotransferrin)	10.100	0.971 − 0.005	0.00E + 00
NK cells (16, 18)	*BSG* (basigin)	6.110	0.659 − 0.038	0.00E + 00
CD56^bright^ NK cells (16)	*GST* (glutathione S‐transferase)	7.303	0.602 − 0.020	0.00E + 00
CD56^dim^ NK cells (18)	*PDCL2* (phosducin‐like 2)	9.114	1.000 − 0.010	0.00E + 00
NKT cells	*ENSMUNG00000003027*	5.456	0.214 − 0.007	1.26E − 32

The B‐cell population was more diverse, resulting in a less optimal general B‐cell marker, being the B lymphocyte transcription factor *BCL11A*, which was only expressed by 52.2% of the B cells. *HVCN1* was identified as a naïve B‐cell marker and has formerly been reported to define other mammalian naïve B‐cell populations. *HVCN1* functions by modulating BCR signal strength via regulation of BCR‐dependent generation of reactive oxygen species [75]. *UHRF1* and *TXNDC5* were selected as GC B‐cell and plasma cell markers and are both known mammalian markers. *TXNDC5*, specifically, plays an important role in enhancing the secretory capacity of mammalian plasma cells [36, 76]. It was not possible to select a suitable marker for memory B cell clusters 3 and 14. The most fit marker (*ND1*) was a mitochondrial gene that was still expressed by 98.6% of all nontargeted cells. This makes us question whether populations 3 and 14 indeed consisted of memory B cells, as these clusters were mostly characterized by an increased expression of mitochondrial genes and a decreased expression of ribosomal proteins. As this has not been reported in avian leukocyte populations before, both clusters remained annotated as memory B cells.

The chemoattractant cytokine *CXCL8* was identified as the general phagocyte marker, expressed by 78.5% of the phagocytes. In mammals, its function is to attract and activate neutrophils in inflammatory regions [77]. In chickens, CXCLi1 and CXCLi2 serve as orthologues of human CXCL8 and perform the same function[49]. *C1QA*, *SPIC*, and *LTF* were defined as markers of M2 macrophages, red pulp macrophages, and heterophils, respectively, and are also known markers for their mammalian counterparts [62, 78, 79]. According to our knowledge, other identified phagocyte markers (*CD9*, *CSF3R*, and *TAGLN3*) have not been associated with M1 macrophages, cDCs, and pDCs before.

NK cells were marked by the expression of *BSG* (also known as *CD147*), important for tumor progression and invasion in mammals. Although expression of *BSG* has been associated with mammalian NK cells before, *GST* and *PDCL2* are newly defined markers, selected for marking CD56^bright^ and CD56^dim^ NK cell populations, respectively [80]. Lastly, the best NKT cell markers were long noncoding RNA fragments (lncRNAs), including *ENSMUNG00000003027*. LncRNAs have been associated with the activation of NKT cells, but their importance was also demonstrated in other T cells. No studies elucidate the function of these lnRNAs in avian T cells yet, but they are believed to have a regulatory function similar to lnRNAs in mammals [81].

### 3.3. Multiplex RNA ISH

Nine cell cluster markers, identified with scRNA seq, were selected, and multiplex RNA ISH was used to validate their expression and provide spatial insight into their cell organization. Additionally, RNA‐ISH was combined with hematoxylin and eosin (H&E) staining to provide knowledge on splenic morphology in psittacines.

**Figure 6:**
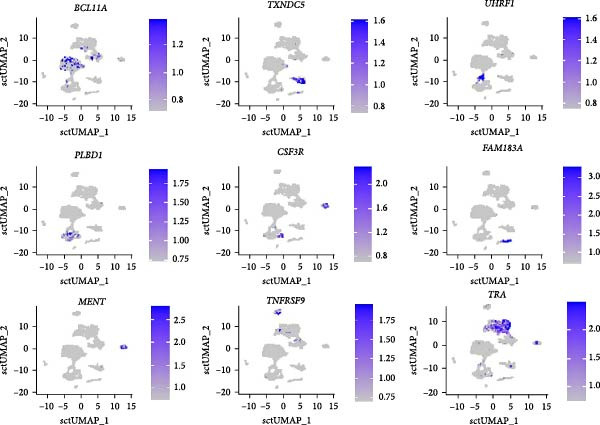
Feature plots of gene markers selected for multiplex RNA‐ISH.

Before multiplex RNA‐ISH was performed, the RNAscope technology was validated on splenic FFPE sections from budgerigars, targeting the housekeeping gene *RPS7*. After adaptation of the pretreatment conditions, RNAscope proved to be fit for use on budgerigar spleen sections. Results of this validation can be found in Figure S2. After validation, following splenic leukocyte population markers were selected for multiplex RNA‐ISH: *BCL11A* (B‐cell marker), *UHRF1* (GC B‐cell marker), *TXNDC5* (plasma cell marker), *TRA* (Th cell marker), *MENT* (heterophil cell marker), *TNFRSF9* (Tcyt marker), *CSF3R* (cDC marker), *PLBD1* (macrophage marker), and *FAM183A* (NK cell marker). *PLBD1* was selected as a general macrophage marker as it selectively marked cell clusters 7, 10, and 21 (avg_log2FC = 5.859, pct.1 = 0.658, pct.2 = 0.026). Further, *MENT* was selected as a heterophil cell marker (avg_log2FC = 11.072, pct.1 = 0.920, pct.2 = 0.003), instead of *LTF*, as it allowed easier probe development and exhibited similar differential expression metrics. The same accounted for the NK cell marker *FAM183A* (avg_log2FC = 8.338, pct.1 = 0.573, pct.2 = 0.028), which replaced *BSG*. Feature plots of these nine markers are visualized in Figure 6.

Unfortunately, the interpretation of the multiplex RNA‐ISH results was complicated by the high level of autofluorescence observed in the budgerigar spleen. Despite efforts to reduce this autofluorescence, using intensive LED light bleaching combined with an autofluorescence‐reducing agent supplied by the RNAscope Hiplex kit manufacturer, the autofluorescence persisted, especially in the AF488 channel, as illustrated in Figure S3. The pronounced autofluorescence was presumably caused by the high amount of red blood cells in the spleen, which are known to exhibit autofluorescence across both red and green wavelengths. Consequently, we were not able to obtain a signal from the markers *FAM183* (NK cells), *CSF3R* (cDCs), and *MENT* (heterophils), all three situated in the AF488 channel. These markers were therefore not included in the subsequent analysis. For future studies, we recommend either labeling these probes with alternative fluorophores or employing additional autofluorescence suppression agents, such as the TrueBlack Lipofuscin Autofluorescence quencher from Biotium, following the approach described by Whittington and Wray [82].

**Figure 7:**
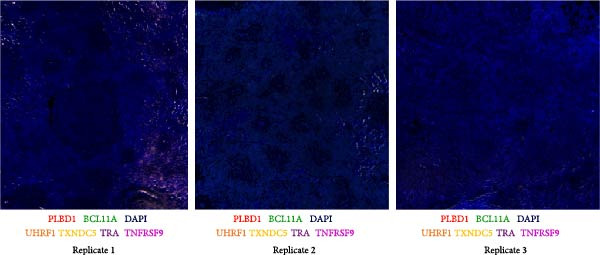
Negative controls of multiplex RNA‐ISH after background subtraction. Residual signal observed after background correction was predominantly concurrent across multiple channels.

The process of subtracting background signal carries the risk of generating false‐positive signals. To address this concern, negative control samples, which lacked probes, were included in the analysis, and background subtraction was applied using the DAPI channel to make the overlay between visualization rounds. The outcome is depicted in Figure 7, revealing the occurrence of a small level of false‐positive signal after background subtraction. Notably, this signal was distributed across multiple channels simultaneously. Furthermore, the intensity of the false‐positive signal was significantly lower in comparison to the positive signal, enabling a clear distinction between false and true signals.

Next, T‐cell markers, B‐cell markers, and the macrophage marker were separately visualized after background subtraction and are presented in Figure 8.

Figure 8RNA‐ISH on budgerigar spleen sections after background subtraction: (A) analysis of T‐cell markers, (B) analysis of B‐cell markers, and (C) analysis of macrophage marker.

**Figure:**
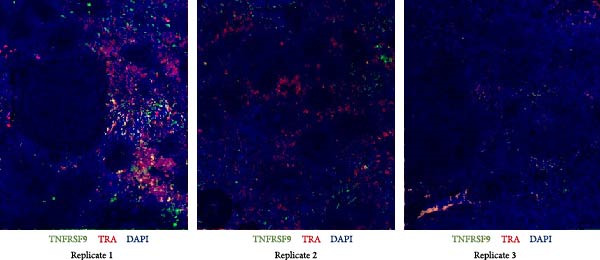
(A)

**Figure:**
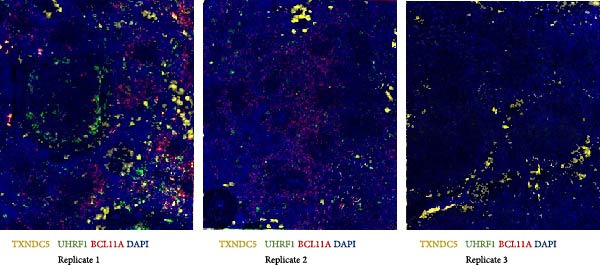
(B)

**Figure:**
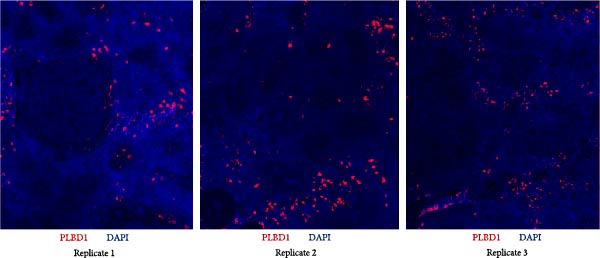
(C)

Following background subtraction, the visualization of B‐cell markers *BCL11A* (B cells), *UHRF1* (GC B cells), and *TXNDC5* (plasma cells) proved successful. In both replicate 1 and 2, *BCL11A*+ cells (B cells) were observed around capillaries, and *UHRF1*+ cells (GC B cells) were predominantly located inside GC, with few cells scattered throughout the spleen. The absence of distinguishable *BCL11A*+ cells (B cells) and *UHRF1*+ cells (GC B cells) in replicate 3 was likely a result of the immunological state of the third budgerigar. Whereas the first and second spleen contained multiple GC, these were absent in replicate 3. *TXNDC5*+ cells (plasma cells) were consistently present in all three replicates, primarily around capillaries and in sinuses.

The presence of *TRA*+ cells (Th cells) was evident in both replicate 1 and 2, situated around arteries, but these cells were almost completely absent in replicate 3. *TNFRSF9* + cells (Tcyt cells) were present in low numbers in all three replicates.

Lastly, *PLBD1*+ cells (macrophages) were observable in all three replicates. Although macrophages were expected to be present in a ring around the B cell region, they were mostly found scattered throughout the red pulp and sparsely distributed within T and B cell regions.

Finally, all markers were consolidated into a single figure (Figure 9), and their localization was elucidated through subsequent H&E staining.

Figure 9RNA‐ISH on budgerigar spleen sections after background subtraction of replicate 1 (A), 2 (B), and 3 (C). On the right side of each replicate, an H&E stain from the same location is presented. White arrow = T cell zone, GC = germinal center, CA = central artery, PC = penicillar capillary, black arrowheads = periarteriolar lymphoid sheath (T cell region), and empty arrowheads = periellipsoid white pulp (B cell region).

**Figure:**
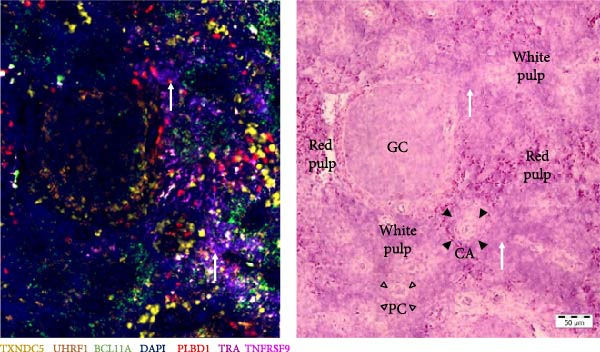
(A)

**Figure:**
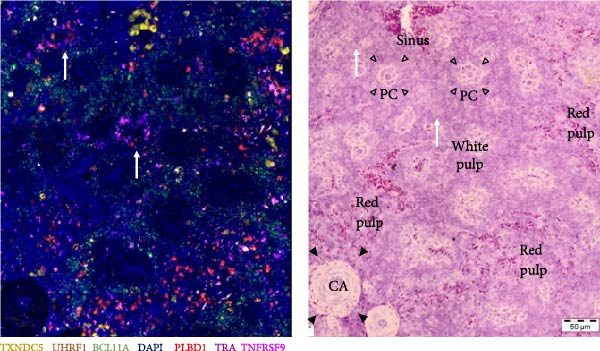
(B)

**Figure:**
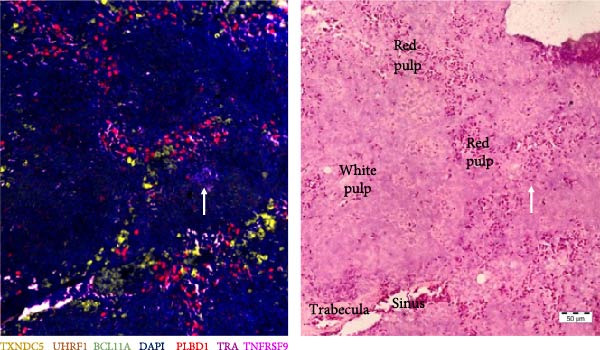
(C)

Figure 9 provides a detailed view of replicate 1, focusing on a GC, characterized by the presence of GC B cells (*UHRF1*+). Contrary to expectations, Th cells (*TRA*+) were not present in the PALS but were dispersed throughout both white and red pulp regions, while B cells (*BCL11A*+) were predominantly located in the PWP region. Plasma cells (*TXNDC5*+) were found around GC, central arteries, and in the red pulp. Similarly, macrophages (*PLBD1*+) were present around GC and central arteries but were mostly located in the red pulp. This is remarkable, as according to the feature plot in Figure 6, *PLBD1* is barely expressed by red pulp macrophages but highly expressed in M1 and M2 macrophages. However, these feature plots were constructed after a scRNA seq analysis on one replicate at one timepoint. The incorporation of multiple replicates during scRNA seq analysis might alter expression levels in these cells. Lastly, a few Tcyt cells (*TNFRSF9*+) colocalized with Th cells (*TRA*+), but overall, only very few *TNFRSF9*+ cells were present.

Although no GC was present in replicate 2 (Figure 9), cell type localization was comparable. B cells (*BCL11A*+) were predominantly found in PWP regions, and Th cells (*TRA*+) were scattered throughout the spleen. Notably, two distinct Th cell (*TRA*+) zones were observed, but not around central arteries. Plasma cells (*TXNDC5*+) were mainly localized in the sinus, and macrophages (*PLBD1*+) were mostly present in red pulp areas.

Replicate 3 (Figure 9), distinct in its immunological state, lacked PWP regions and GCs. Consequently, B cells (*BCL11A*+) and GC B cells (*UHRF1*+) were scarce. Few Th (*TRA*+) and Tcyt cells (*TNFRSF9*+) were visualized, with one clear T‐cell zone present in the white pulp. Macrophages (*PLBD1*+) were more abundant in this replicate and were primarily located in the red pulp.

From the nine markers that were analyzed, six markers could be visualized. The B cell markers were mostly located as described in earlier avian morphological studies. GC B cells were found in GC, whereas plasma cells were located in the red pulp, around arteries, and GC. General B cells were mainly localized around penicillar capillaries, in the PWP, representing the equivalent of the mammalian marginal zone [1]. The localization of Th and Tcyt cells was more remarkable. Earlier studies described their presence mainly in the PALS, with few single T cells scattered throughout the red pulp [1, 13]. In this study, Th and Tcyt cells were situated in distinct regions within the white pulp, separate from both the B cell region and the PALS. The location of the macrophage marker was surprising as well. According to the scRNA seq analysis, *PLBD1* is predominantly expressed by M1 and M2 macrophages, not by red pulp macrophages. Nevertheless, its expression was more pronounced in red pulp. Further, Liman and Bayram [10] reported the presence of a progressive number of macrophages around ellipsoids in quails, something that was completely absent in these budgerigar spleens [10].

Although the different features of these markers were not completely elucidated, they did provide additional information on the structure and organization of the spleen. Nevertheless, further examination is necessary before their use in immunological assays.

## 4. Conclusion

In this study, a cell‐type annotated reference single‐cell dataset of budgerigar splenocytes was constructed. Although many similarities were found between chicken spleen leukocytes and budgerigar spleen leukocytes, a few differences indicated the necessity for species‐specific analysis. Among these differences, the complete absence of the well‐known leukocyte marker CD8β and classical immunoglobulin genes were perhaps most remarkable. For each of the annotated splenic leukocyte populations, markers were defined, and nine of them were selected for validation with multiplex RNA‐ISH. During RNA‐ISH, splenic morphology was analyzed, and six markers were validated according to their localization in the spleen. The other three markers could not be validated due to the high level of autofluorescence. In contradiction to other studied avian spleens, T cells were not concentrated around central arteries, although distinct T‐cell zones were distinguishable in the white pulp. On the other hand, B cells were found in PWP, representing the counterpart of the mammalian marginal zone. This study demonstrated the potential of an immunological toolbox consisting of scRNA seq and RNA‐ISH to study the immune system when other immunological assays are lacking/unavailable. However, it is important to emphasize that the scRNA seq analysis was based on a single biological replicate at one time point, which limits the generalizability of the findings. Therefore, future studies should incorporate additional biological replicates across multiple time points to strengthen the robustness and reproducibility of these initial observations.

## Ethics Statement

The research was evaluated and approved by the Ethical Committee for Animal Experiments of Ghent University (Reference Number: EC‐2019/086).

## Conflicts of Interest

The authors declare no conflicts of interest.

## Author Contributions

**Anne De Meyst**: methodology, formal analysis, writing – original draft. **Kelly Lemeire**: methodology, writing – review and editing. **Amanda Gonçalves**: methodology, writing – review and editing. **Niels Vandamme**: methodology, writing – review and editing. **Daisy Vanrompay**: writing – review and editing, supervision.

## Funding

This work was supported by the Research Foundation Flanders (FWO) (Grant 1S67121N).

## Supporting Information

Additional supporting information can be found online in the Supporting Information section.

## Supporting information


**Supporting Information** Table S1: List of gene abbreviations. Figure S1: Raw image of heatmap generated with R version 4.1.2. Figure S2: Validation of RNAscope RED technology on budgerigar splenic FFPE sections: (A) RPS7 feature plot, (B) RNAscope RED positive control, (C) RNAscope RED negative control, and (D) RNAscope RED sample, showing RPS7 expression. Figure S3: Whole‐slide images of the three budgerigar spleens in visualization round 1 (signal) and round 4 (background). For both rounds, signal in the DAPI (blue), AF488 (green), Dylight 550 (red), and Dylight 650 (purple) channels is shown. This figure demonstrates the high level of autofluorescence, which is notably the strongest in the AF488 channel.

## Data Availability

The data discussed in this publication have been deposited in NCBI’s Gene Expression Omnibus [83] and are accessible through GEO Series accession number GSE273701 (https://www.ncbi.nlm.nih.gov/geo/query/acc.cgi?acc=GSE273701).
