# Supplementary material for: Unmasking Budgerigar Splenic Leukocyte Populations With Single‐Cell Transcriptomics and Multiplex RNA In Situ Hybridization
Source: J Immunol Res. 2025 Oct 9;2025:1554863. doi: 10.1155/jimr/1554863 (PMC12511316; doi:10.1155/jimr/1554863)
Supplement: Supplementary file 1 — Supporting Information Table S1: List of gene abbreviations. Figure S1: Raw image of heatmap generated with R version 4.1.2. Figure S2: Validation of RNAscope RED technology on budgerigar splenic FFPE sections: (A) RPS7 feature plot, (B) RNAscope RED positive control, (C) RNAscope RED negative control, and (D) RNAscope RED sample, showing RPS7 expression. Figure S3: Whole‐slide images of the three budgerigar spleens in visualization round 1 (signal) and round 4 (background). For both rounds, signal in the DAPI (blue), AF488 (green), Dylight 550 (red), and Dylight 650 (purple) channels is shown. This figure demonstrates the high level of autofluorescence, which is notably the strongest in the AF488 channel. [file JIMR-2025-1554863-s001.docx]

Supplementary Material

# Supplementary Figures and Tables

## Supplementary Figures

**Supplementary Figure S.1:** Raw image of heatmap generated with R version 4.1.2.


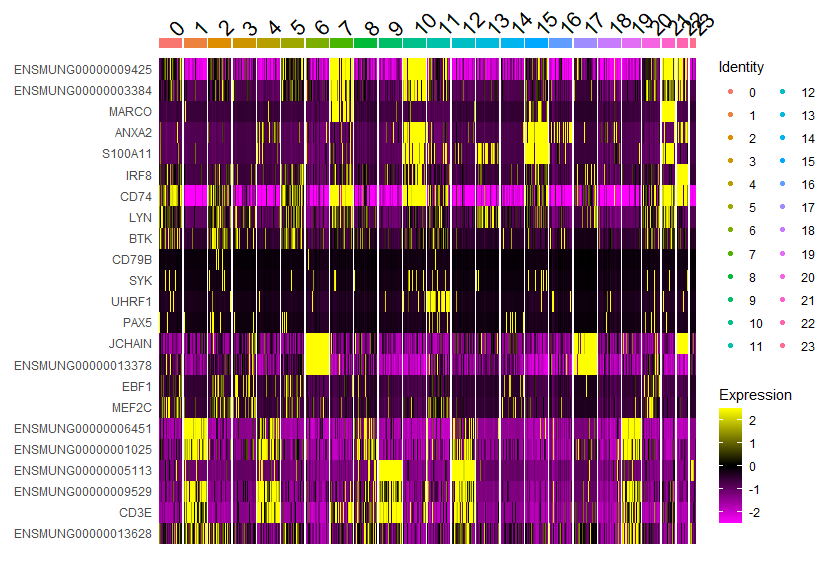


**Supplementary Figure S.2:** Validation of RNAscope® RED technology on budgerigar splenic FFPE sections: (A) *RPS7* feature plot, (B) RNAscope® RED Positive Control, (C) RNAscope® RED Negative Control and (D) RNAscope® RED sample, showing RPS7 expression.


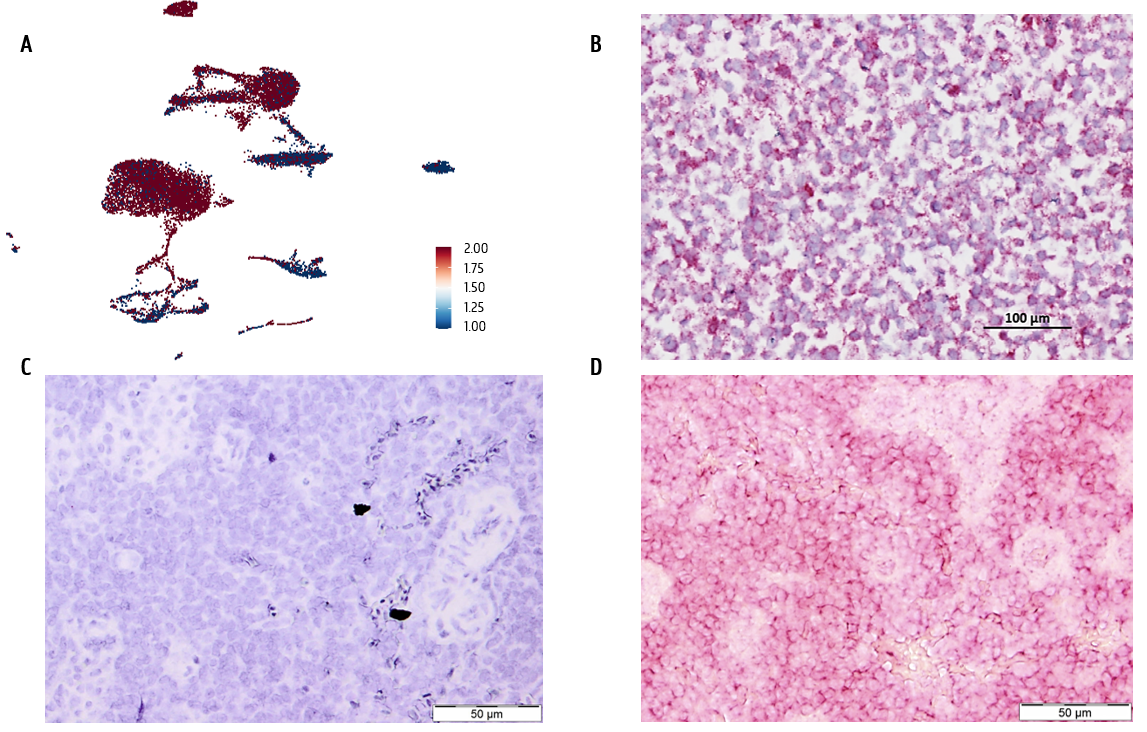


**Supplementary Figure S.3:** Whole-slide images of the three budgerigar spleens in visualisation round 1 (signal) and round 4 (background). For both rounds, signal in the DAPI (blue), AF488 (green), Dylight 550 (red), and Dylight650 (purple) channel is shown. This figure demonstrates the high level of autofluorescence, which is notably the strongest in the AF488 channel.


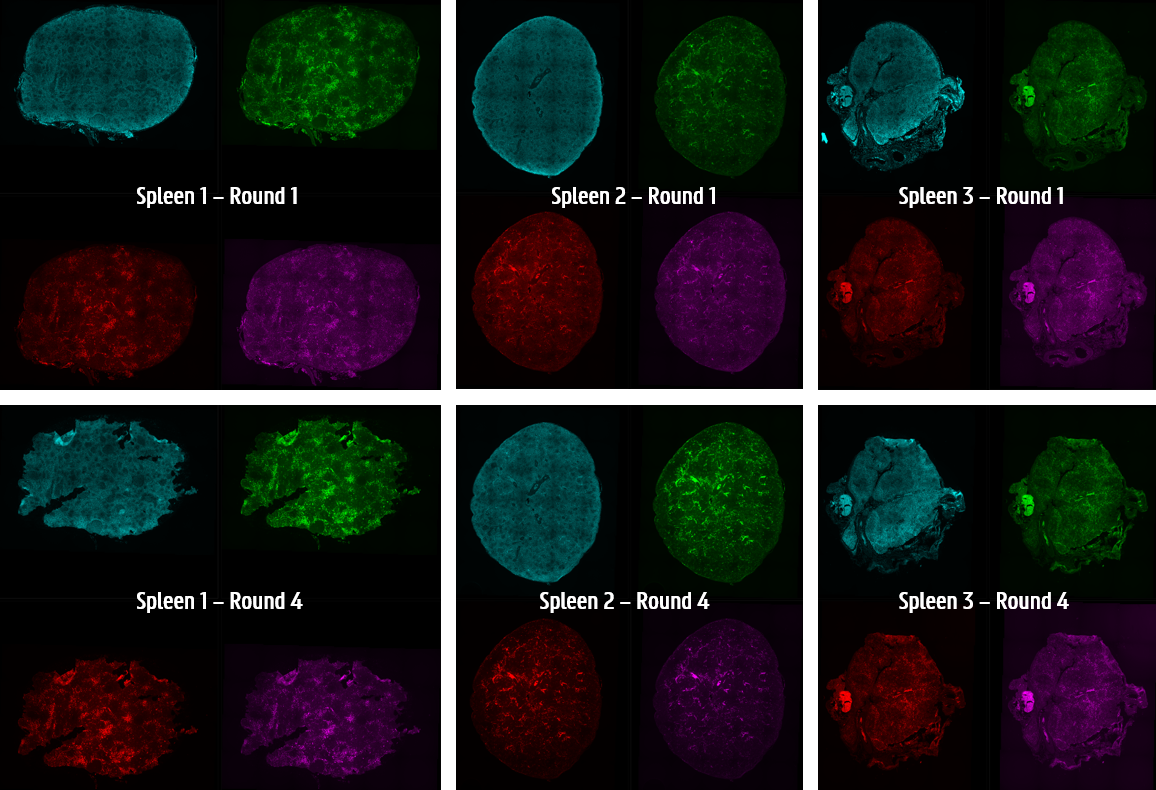


## Supplementary Tables

**Supplementary Table S.1**: List of gene abbreviations

| Gene | Complete Gene Name |
| --- | --- |
| TRA | T cell receptor α chain |
| TRB | T cell receptor β chain |
| TRD | T cell receptor δ chain |
| CD3ε | Cluster of differentiation 3ε |
| CD3γ | Cluster of differentiation 3δ |
| MHC-DRA | Major histocompatibility complex, class II, DRα |
| MHC-DRB1 | Major histocompatibility complex, class II, DRβ1 |
| MARCO | Macrophage receptor with collagenous structure |
| ANXA2 | Annexin A2 |
| S100A11 | S100 calcium-binding protein A11 |
| KLF2 | Krüppel-like factor 2 |
| IRF8 | Interferon regulatory factor 8 |
| CD74 | Cluster of differentiation 74 |
| LYN | LYN Proto-Oncogene, Src Family Tyrosine Kinase |
| BTK | Bruton's tyrosine kinase |
| CD79b | Cluster of differentiation 79b |
| Syk | Spleen tyrosine kinase |
| JCHAIN | Joining chain of multimeric IgA and IgM |
| IGLL1 | Immunoglobulin λ-like polypeptide 1 |
| UHRF1 | Ubiquitin-like with PHD and ring finger domains 1 |
| Pax5 | Paired box 5 |
| EBF1 | Early B-cell factor 1 |
| MEF2C | Myocyte enhancer factor 2C |
| CD4 | Cluster of differentiation 4 |
| CD8α | Cluster of differentiation 8α |
| CD8β | Cluster of differentiation 8β |
| XCL1 | X-C Motif Chemokine Ligand 1 |
| TNFRSF9 | Tumour necrosis factor receptor superfamily member 9 |
| IL21R | Interleukin 21 receptor |
| Eomes | Eomesodermin |
| GATA-3 | GATA-binding protein 3 |
| IFT5 | Interferon Induced Protein with Tetratricopeptide Repeats 5 |
| LY6E | Lymphocyte antigen 6E |
| MX1 | MX Dynamin Like GTPase 1 |
| IF27L2 | Interferon, α-inducible protein 27-like 2 |
| STAT1 | Signal transducer and activator of transcription 1 |
| IL-4 | Interleukin 4 |
| IL-13 | Interleukin 13 |
| IL-17 | Interleukin 17 |
| FoxP3 | Forkhead box P3 |
| IL18R1 | Interleukin 18 receptor 1 |
| ICOS | Inducible T-cell costimulator |
| CCR6 | C-C motif chemokine receptor 6 |
| TCF7 | Transcription factor 7 |
| TNFRSF18 | Tumour necrosis factor receptor superfamily member 18 |
| HMGN5 | High mobility group nucleosome-binding domain 5 |
| SMC2 | Structural Maintenance of Chromosomes 2 |
| STMN1 | Stathmin 1 |
| CREB3L2 | CAMP responsive element binding protein 3-like 2 |
| RPS11 | Ribosomal protein S11 |
| UQCRQ | Ubiquinol-cytochrome c reductase complex III subunit VII |
| GTPB4 | GTP binding protein 4 |
| COX7A2 | Cytochrome c oxidase subunit 7A2 |
| MYC | MYC Proto-Oncogene, BHLH Transcription Factor |
| SPIC | Spi-C Transcription Factor |
| CCR6 | C-C motif chemokine receptor 6 |
| CD206 | Cluster of differentiation 206 |
| C1QA | Complement c1q A chain |
| C1QB | Complement c1q B chain |
| C1QC | Complement c1q C chain |
| DCSTAMP | Dendritic cell-specific transmembrane protein |
| TLR3 | Toll-like receptor 3 |
| CD2AP | CD2 Associated Protein |
| NCAM1 (CD56) | Neural Cell Adhesion Molecule 1 |
| RUNX3 | Runt-related transcription factor 3 |
| CXCL8 | CXC motif chemokine ligand 8 |
| HMGB1 | High mobility group box 1 |
| SPI1 | Spi-1 Proto-Oncogene |
| HCVN1 | Hydrogen voltage-gated channel 1 |
| TXNDC5 | Thioredoxin domain-containing 5 |
| ND1 | NADH dehydrogenase 1 |
| LTF | Lactotransferrin |
| CD9 | Cluster of differentiation 9 |
| CSF3R | Colony-stimulating factor 3 receptor |
| TAGLN3 | Transgelin 3 |
| GST | Glutathione S-transferase |
| PDCL2 | Phosducin-like 2 |
